# Detection of Organophosphorus Pesticides in Wheat by Ionic Liquid-Based Dispersive Liquid-Liquid Microextraction Combined with HPLC

**DOI:** 10.1155/2018/8916393

**Published:** 2018-05-02

**Authors:** Wei Liu, Ji Quan, Zeshu Hu

**Affiliations:** ^1^School of Resources and Environmental Engineering, Wuhan University of Technology, No. 122 Luoshi Road, Wuhan 430070, China; ^2^School of Management, Wuhan University of Technology, No. 122 Luoshi Road, Wuhan 430070, China

## Abstract

Food safety issues closely related to human health have always received widespread attention from the world society. As a basic food source, wheat is the fundamental support of human survival; therefore, the detection of pesticide residues in wheat is very necessary. In this work, the ultrasonic-assisted ionic liquid-dispersive liquid-liquid microextraction (DLLME) method was firstly proposed, and the extraction and analysis of three organophosphorus pesticides were carried out by combining high-performance liquid chromatography (HPLC). The extraction efficiencies of three ionic liquids with bis(trifluoromethylsulfonyl)imide (Tf_2_N) anion were compared by extracting organophosphorus in wheat samples. It was found that the use of 1-octyl-3-methylimidazolium bis(trifluoromethylsulfonyl)imide ([OMIM][Tf_2_N]) had both high enrichment efficiency and appropriate extraction recovery. Finally, the method was used for the determination of three wheat samples, and the recoveries of them were 74.8–112.5%, 71.8–104.5%, and 83.8–115.5%, respectively. The results show that the method proposed is simple, fast, and efficient, which can be applied to the extraction of organic matters in wheat samples.

## 1. Introduction

Food cultivation is the main part of agricultural production. In the process of agricultural cultivation, pesticide spraying is the dominant approach to protect the healthy growth of plants. Organophosphorus pesticides (OPPs), as an inexpensive, stable, and efficient pesticide, are usually and widely used in agricultural production in the world [1]. OPPs can inhibit the activity of acetylcholinesterase, and acetylcholine in body is thereby accumulated, which can have a serious effect on central nervous system, can cause symptoms of poisoning, and can even lead one to death. Because of their toxicity and abuse, the pollution of water and land by OPPs has also become a serious environmental problem, which at all times threatens people's lives. As the main and basic food crop for human beings, wheat is the source of daily food that people often come into contact with. The pesticide residue in wheat must be controlled and monitored. Therefore, in order to ensure food safety and human health, the detection of OPPs in wheat is very necessary [2].

In the pesticide residue analysis, the commonly used detection methods are mainly gas chromatography (GC) [3], gas chromatography-mass spectrometry (GC-MS) [4], high-performance liquid chromatography (HPLC) [5], and liquid chromatography tandem mass spectrometry (LC-MS/MS) [6]. Although LC-MS/MS and GC-MS show excellent detection capability, the high cost still inhibits their widespread use. Compared to them, HPLC with its convenience, efficiency, and durability, is the most extensive means of pesticide detection.

The traditional methods of extraction of pesticides in wheat are mainly liquid-phase extraction (LLE) [7], liquid-phase microextraction (LPME) [8], supercritical fluid extraction (SFE) [9], and so on. In recent years, a rapid, simple, and convenient dispersive liquid-liquid microextraction (DLLME) [10] method has been proposed and then is rapidly applied to various drug extraction studies, especially in the field of pesticide extraction. In the process of DLLME, the extractant, dispersant, and water form a three-phase system, and the analytes and the extractant are deposited at the bottom of the centrifuge tube by centrifugation, followed by quantitative analysis by means of analytical instruments; the whole process is very simple and efficient, thus, it is a promising method for trace extraction. In the traditional DLLME, the commonly used extractants are chlorobenzene [11], dichloromethane [12], dibromoethane [13], and so on. They are toxic, hazardous, flammable, and environmentally damaging organic solvents and difficult to reuse [14]. The use of green, low-toxic, and highly-efficient extractants is an inevitable trend for DLLME.

As a class of green solvent, ionic liquids (ILs) are gaining huge attention since their unique properties especially negligible vapour pressure, wide range of solubility, miscibility, and stability at high temperatures; they are good replacements for conventional volatile and toxic organic solvents in chemical processes [15]. One of the advantages arising from the chemical structures of ILs is that alteration of the cation or anion can cause changes in properties such as viscosity, melting point, water miscibility, and density [14]. Due to the ILs often showing great capability of dissolving both organic and nonorganic compounds, it is easy to separate an IL from the reaction system [16], the application of ILs in DLLME is getting a growing interest in drugs and pesticides extraction in analytical chemistry [17–19].

ILs with 1-alkyl-3-methylimidazolium cation and anions of bromide (Br^−^), hexafluorophosphate (PF_6_^−^) and bis(trifluoromethylsulfonyl)imide (Tf_2_N^−^) are most commonly used as extraction solvents in many literatures. For example, 1-butyl-3-methylimidazolium hexafluorophosphate ([BMIM][PF_6_]) was used to extract benzodiazepines [20], 1-hexyl-3-methylimidazolium bis(trifluoromethylsulfonyl)imide ([HMIM][Tf_2_N]) was used to extract bisphenol A [21], 1-hexyl-3-methylimidazolium hexafluorophosphate ([HMIM][PF_6_]) was used to extract hexachlorophene [22], and 1-butyl-3-methylimidazolium bromide ([BMIM] Br) was the extraction solvent of brazilin and protosappanin B [23]. The ILs showed good extraction efficiency in their extraction systems.

There are two factors influencing the solubility and miscibility of a given ionic liquid. One is the type of its anion and the other is the length of the alkyl chain of the cation. As literature reported [15], increase of the alkyl chain causes increase of the capacity and hydrophobicity of the ILs, and the bigger the anion size, the stronger the hydrophobicity. The size of Tf_2_N anion is bigger than PF_6_^−^ and BF_4_^−^, thus, the Tf_2_N anion-based ILs exhibit high hydrophobicity and capacity, which with the same effect will save the use of IL volume. In addition, the strong delocalization and diffuse nature of the negative charge in the S–N–S core of [Tf_2_N]^−^ leads to a reduction in cation-anion interactions [24]. Due to these properties, Tf_2_N anion-based ILs have attracted broad attention and have been widely applied in chemical processes such as recovery of metal [25], organics extraction [26], and CO_2_ capture [27], and they are promising extraction agents for pesticides extraction.

Although there are many literatures employing Tf_2_N anion-based ILs and DLLME for metal ion extraction [19], the application of them for OPPs extraction is rare. Furthermore, IL-DLLME for pesticide extraction is often applied in water or liquid samples. As far as we know, there is no report about using Tf_2_N anion-based ILs as extraction solvent in the DLLME process to extract OPPs from wheat yet.

Understanding of the structure and features of Tf_2_N-based ILs is of great interest due to their exclusive physicochemical properties. This work compared the OPPs extraction capacity of ILs with Tf_2_N anion and three different cations ([HHIM], [OMIM], and [BeOIM]) in wheat samples for the first time, and applied the ILs in the DLLME method followed by HPLC analysis. Different factors influencing the extraction efficiency including the extractant type and volume, dispersant type and volume, and temperature were investigated. The proposed method in this work fills the gap in the extraction method of pesticide residues in wheat samples by Tf_2_N-based ILs.

## 2. Experimental

### 2.1. Reagents, Standards, and Materials

**Figure 1:**
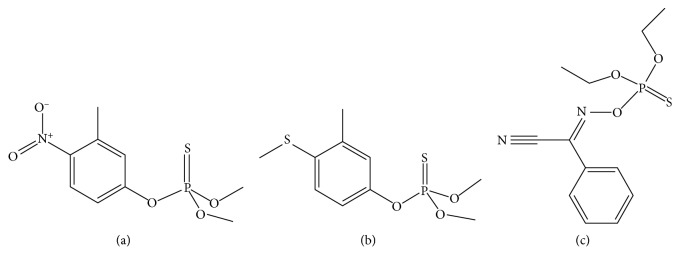
Structures of three OPPs. (a) Fenitrothion. (b) Fenthion. (c) Phoxim.

Organophosphorus pesticides (OPPs) of fenitrothion, fenthion, and phoxim were purchased from Beijing Agricultural Environmental Protection Center, the structure of them are shown in Figure 1. Acetonitrile and methanol (HPLC grade) were obtained from Tianjin Siyou Fine Chemicals Co., Ltd. (Tianjin, China). The 1-octyl-3-methylimidazole bis(trifluoromethylsulfonyl)imide ([OMIM][Tf_2_N]), 1,3-dihexylimidazole bis(trifluoromethylsulfonyl)imide ([HHIM][Tf_2_N]), and 1-benzyl-3-octylimidazole bis(trifluoromethylsulfonyl)imide ([BeOIM][Tf_2_N]) were laboratory made. Standard solutions with the concentration of 1 mg/ml were prepared by dissolving each OPP standard (0.0100 g) into 10.0 mL acetonitrile and stored at 4°C. Wheat samples were purchased from Henan Agricultural Sciences Institute (Zhengzhou, Henan).

### 2.2. Apparatus

HPLC analysis was carried out by the Shimadzu HPLC system which was equipped with an LC-10AT pump (Shimadzu, Japan), an SPD-10A UV-VIS detector (Shimadzu, Japan), a Shimadzu VP-ODS column (150 mm × 4.6 mm i.d., 5 *μ*m), and a Rheodyne 7725i six-way valve injector with 20 *μ*L sample loop (Rheodyne, Rohnert Park, CA, USA). The mobile phase was the mixture of methanol and water (70 : 30, *v*/*v*) with the flow rate of 1 mL/min. The wavelength was set at 254 nm. A JP010/S ultrasonic cleaner (Shenzhen Jie UNITA Cleaning Equipment Co., Ltd., Shenzhen, China) was used for extracting OPPs from wheat sample into the acetonitrile phase. An 80-1 centrifuge (Huafeng Instrument Co. Ltd., Jintan, China) was used for centrifuging.

### 2.3. Extraction Procedure

#### 2.3.1. Wheat Sample Extraction (Step 1)

Wheat samples were grinded into fine powder, and 1 g powder was placed in a 10 mL centrifuge tube, then a solution of 5 mL methanol containing 110 *μ*L [OMIM][Tf_2_N] was added into the centrifuge tube and followed by ultrasonic treatment for 8 min.

#### 2.3.2. DLLME Procedure (Step 2)

After centrifugation for 5 min, the obtained methanol solution containing OPPs and ionic liquid was taken out into a centrifuge tube including 5 mL distilled water, and a cloudy solution was formed in the tube. Then, it was centrifuged for 5 min. Finally, 5 *μ*L of [OMIM][Tf_2_N] sedimentary facies formed in the bottom of the centrifuge tube was injected to the HPLC system for analysis.

### 2.4. Enrichment Factor and Extraction Recovery

Enrichment factor (EF) and extraction recovery (ER) were the two evaluating indicators of this developed method. They were calculated by the equation as follows:

(1) $$\begin{array}{c} \begin{array}{l} \text{EF}=\frac{C_{\text{sed}}}{C_{0}},\\ \text{ER}=\frac{C_{\text{sed}}\times V_{\text{sed}}}{C_{0}\times V_{\text{aq}}}\times 100\%=\text{EF}\times \frac{V_{\text{sed}}}{V_{\text{aq}}}\times 100\%. \end{array} \end{array}$$

*C*_0_ and *C*_sed_ express the concentration of the OPPs in the DLLME procedure and the concentration in sedimentary facies, respectively. *V*_aq_ and *V*_sed_ stand for the volume of aqueous solution and sedimentary facies.

Experimental data were the average of three repetitions in each case.

## 3. Results and Discussion

### 3.1. Method Optimization

#### 3.1.1. Selection of Solvent in Ultrasonic Extraction

The selection of the solvent in ultrasonic extraction is very important for the efficient extraction of the target from the wheat sample. In fact, the solvent in ultrasonic extraction plays a dual role in the whole extraction process, and it acts as an extractant for wheat samples and simultaneously as a dispersant in the process of DLLME.

Therefore, the solvent in the ultrasonic extraction needs to satisfy the following conditions:

1. Effective extraction for the targets in wheat
2. Excellent dispersibility for extractants in the process of DLLME

**Figure 2:**
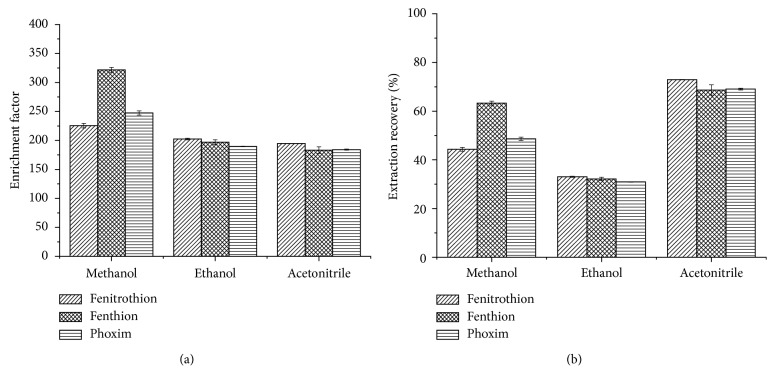
Effects of different reagents on EF (a) and ER (b). Extraction conditions: solvents, methanol, ethanol, and acetonitrile, respectively; solvent volume, 1 mL; extractant, [OMIM][Tf_2_N]; extractant volume, 22 *μ*L; extraction temperature, room temperature; ultrasonic time, 8 min; centrifugal time, 5 min.

In order to select the appropriate solvent, methanol, ethanol, and acetonitrile were investigated experimentally. The results are shown in Figure 2. It can be seen from the figure that when methanol is used as the solvent, the EFs of the three OPPs are the highest, and the ER values are slightly lower than when acetonitrile is used. Although, the ERs of the targets are the highest when acetonitrile is used as the solvent, the EFs are the lowest. When using ethanol, both the EFs and ERs are low. In order to ensure both EFs and ERs are higher, ultimately, methanol was chosen as the solvent in the ultrasonic extraction process, which also acts as a dispersant for the DLLME process.

#### 3.1.2. Selection of Methanol Volume

The choice of methanol volume in the ultrasonic extraction process requires two aspects: on one hand, there is a need for sufficient methanol to facilitate the extraction of the OPPs from the wheat sample as much as possible; on the other hand, a suitable volume of methanol is required to better disperse the ionic liquid in the DLLME to obtain a suitable deposition phase volume for subsequent injection analysis. Therefore, it is necessary to examine the volume of methanol.

**Figure 3:**
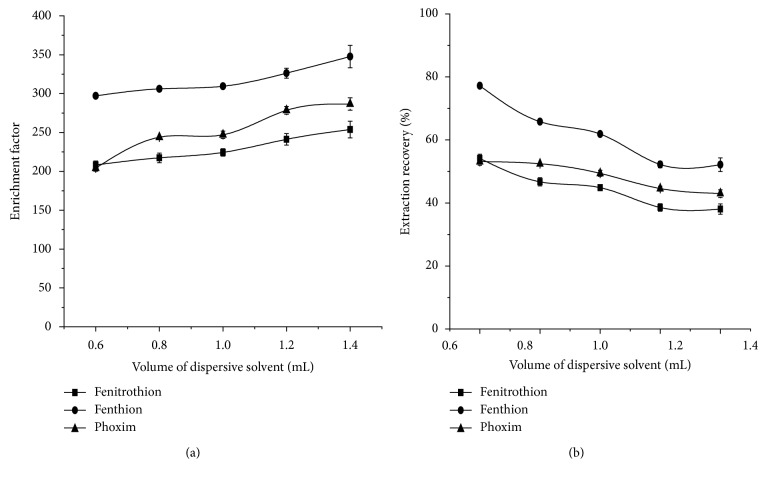
Effect of methanol volume on EFs (a) and ERs (b). Extraction conditions: solvent, methanol; solvent volume, 0.6 mL, 0.8 mL, 1 mL, 1.2 mL, and 1.4 mL, respectively; extractant, [OMIM][Tf_2_N]; extractant volume, 22 *μ*L; extraction temperature, room temperature; ultrasonic time, 8 min; centrifugal time, 5 min.

The effect of extraction on three OPPs was investigated when the volume of methanol was changed from 0.6 to 1.4 mL (containing 22 *μ*L [OMIM][Tf_2_N]). The results are shown in Figure 3.

As can be seen from the figure, with the increase in methanol volume, EFs show an increasing trend, on the contrary, ERs show a downward trend. This can be explained by the fact that the increase in the volume of methanol is better to extract the targets from the wheat sample and that increases the solubility of the extractant (ionic liquid) in the DLLME, thus, decreases the volume of the deposition phase from 13 *μ*L to 7.5 *μ*L, so EFs increase, while ERs become smaller.

Considering the EF and ER values, 1.0 mL of methanol was selected to use.

#### 3.1.3. Selection of Ionic Liquid Species

In this paper, ionic liquid was employed for the extractant in the DLLME process. The choice of ionic liquid type is very important. Because different ionic liquids have different solubilities and extraction abilities, the dispersion effects in aqueous solution are different, which can affect the volume of sedimentary facies and finally affect the extraction results.

**Figure 4:**
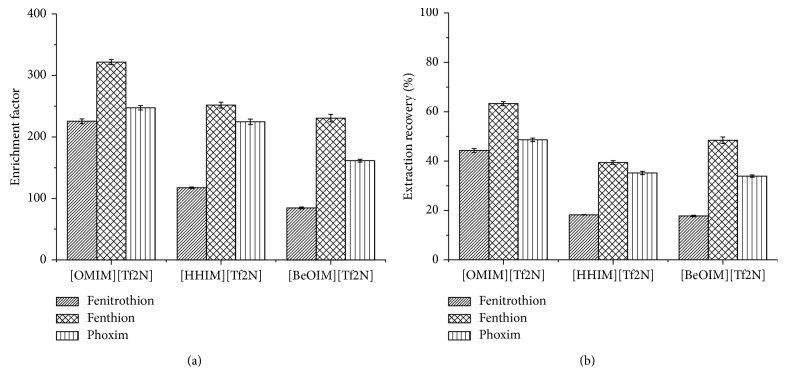
Effects of IL species on EF (a) and ER (b). Extraction conditions: solvent, methanol; solvent volume, 1 mL; extractant, [OMIM][Tf_2_N], [HHIM][Tf_2_N], and [BeOIM][Tf_2_N], respectively; extractant volume, 22 *μ*L; extraction temperature, room temperature; ultrasonic time, 8 min; centrifugal time, 5 min.

In order to select the appropriate IL type, the results of the extraction of three ILs ([OMIM][Tf_2_N], [HHIM][Tf_2_N], and [BeOIM][Tf_2_N], resp.) with the same anions ([Tf_2_N^−^]) were compared using the same volume (22 *μ*L). The results are shown in Figure 4. The figure shows that using [OMIM][TF_2_N] as the extractant has the highest EF and ER values. So [OMIM][TF_2_N] was selected as the DLLME extractant.

#### 3.1.4. Selection of [OMIM][TF_2_N] Volume

**Figure 5:**
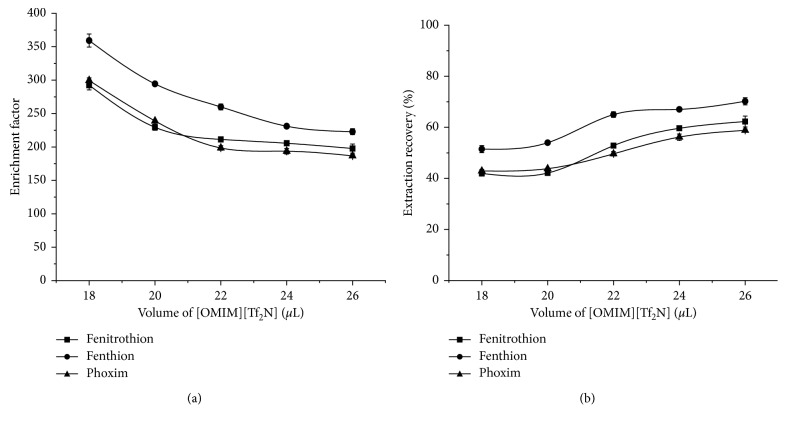
Effects of [OMIM][TF_2_N] volume on EFs (a) and ERs (b). Extraction conditions: solvent, methanol; solvent volume, 1 mL; extractant, [OMIM][Tf_2_N]; extractant volume, 18 *μ*L, 20 *μ*L, 22 *μ*L, 24 *μ*L, and 26 *μ*L, respectively; extraction temperature, room temperature; ultrasonic time, 8 min; centrifugal time, 5 min.

In the DLLME process, the volume of the extractant will affect the volume of the deposited phase and also affect the formation of the dispersant-extractant-water three-phase suspension system, thereby affecting the extraction effect. In order to obtain the optimized amount of IL, the effect of [OMIM][Tf_2_N] volume from 18–26 *μ*L on the extraction results was investigated during the DLLME process. It can be seen from Figure 5 that the EFs of the three OPPs decrease as the volume of [OMIM][TF_2_N] increases while the ERs increase. This is because the larger the [OMIM][TF_2_N] volume was used, the poorer the dispersion effect in the water formed and the larger the volume of the deposited phase, from 7.16 *μ*L to 15.75 *μ*L, was obtained, resulting in a decrease in the EFs and an increase in the ERs. Considering the EFs and ERs, the volume of [OMIM][TF_2_N] was chosen to be 22 *μ*L.

#### 3.1.5. Selection of Ultrasonic Time

**Figure 6:**
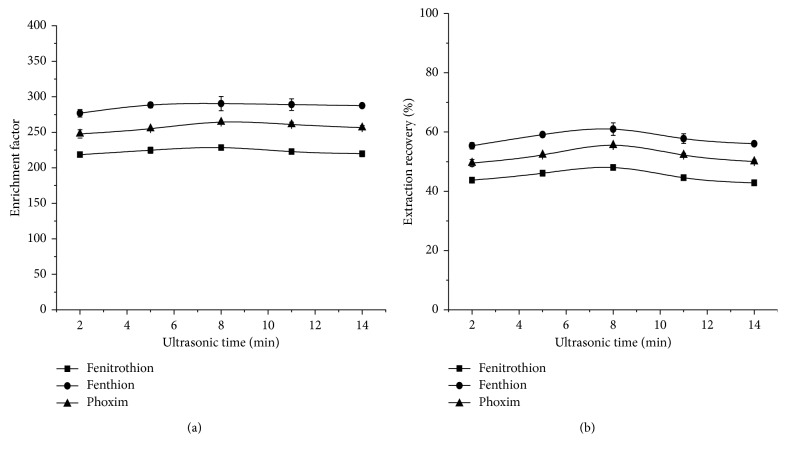
Effects of ultrasonic time on EFs (a) and ERs (b). Extraction conditions: solvent, methanol; solvent volume, 1 mL; extractant, [OMIM][Tf_2_N]; extractant volume, 22 *μ*L; extraction temperature, room temperature; ultrasonic time, 2 min, 5 min, 8 min, 11 min, and 14 min, respectively; centrifugal time, 5 min.

Ultrasonic time will affect the dissolution of OPPs from wheat samples to methanol solution, which will affect the extraction of DLLME and ultimately affect EFs and ERs of the OPPs. The effects of ultrasonic extraction time from 2 min to 14 min on the extraction efficiency were investigated. Results are shown in Figure 6. It can be seen from the figure, when the time of ultrasound is set to 8 min, both EFs and ERs are high, so 8 min was selected as the ultrasonication time.

#### 3.1.6. Selection of Temperature in DLLME

**Figure 7:**
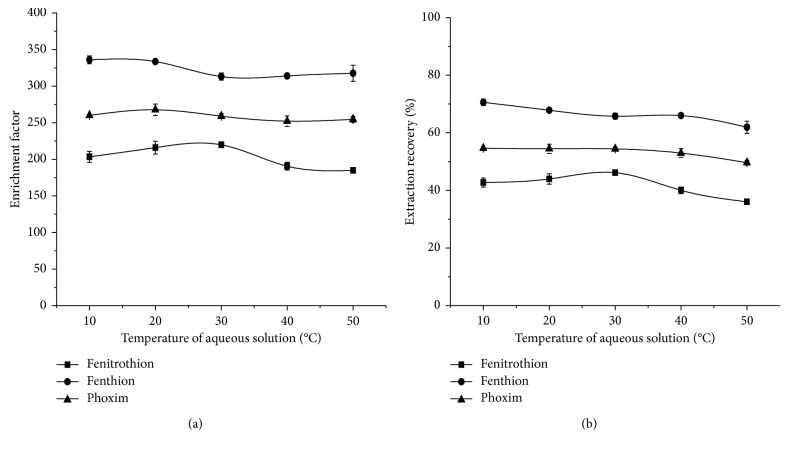
Effect of aqueous solution temperature on EFs (a) and ERs (b). Extraction conditions: solvent, methanol; solvent volume, 1 mL; extractant, [OMIM][Tf_2_N]; extractant volume, 22 *μ*L; extraction temperature, 10°C, 20°C, 30°C, 40°C, and 50°C, respectively; ultrasonic time, 2 min, 5 min, 8 min, 11 min, and 14 min, respectively; centrifugal time, 5 min.

The temperature of DLLME may affect the mass transfer efficiency of OPPs in wheat, thus affecting the extraction results. Therefore, it is necessary to examine the effect of temperature on the extraction efficiency of the targets. The effect of temperature on the extraction efficiency was investigated by changing the temperature of the aqueous solution (10°C–50°C). The results are shown in Figure 7. The figure indicates that when the temperature of the aqueous solution is 20°C, the EFs and ERs of the OPPs are higher; when the temperature is 50°C, the solubility of [OMIM][TF_2_N] increased and the volume of sedimentary facies decreased slightly from 10.5 *μ*L to 9.75 *μ*L, which reduced ERs slightly. Because the DLLME process is very short, extraction can be done in an instant, the overall effect of aqueous solution temperature is not significant for simple experimental operation, and DLLME was carried out at room temperature.

#### 3.1.7. Selection of Centrifugal Time

In the process of DLLME, in order to separate the ionic liquid-phase from the aqueous phase, it is necessary to separate the extractant-dispersant-water three-phase system. The length of the centrifugal time will affect the volume of the deposited phase, resulting in changes in EFs and ERs. In this work, consistent with literature [28], 5 min is selected for centrifugation because 5 min of centrifugation is enough to ensure the deposition of [OMIM][Tf_2_N] ionic liquid owing to its high hydrophobicity.

### 3.2. Evaluation of Method Performance

In order to evaluate the proposed method for extracting three OPPs from the wheat sample, parameters including linearity, repeatability, and limits of detection were obtained and investigated through a series of experiments under the optimized conditions.  Table 1 shows the results. Under optimized conditions, the EFs range from 203.8 to 332.4. The method has a good linear relationship between 0.1 and 100.0 *μ*g/g, and the range of correlation coefficient (*r*^2^) is 0.9973–0.9998. The relative standard deviations (RSDs) were between 0.6% and 6.3% (*n*=5). The limits of detection (LODs), based on a signal-to-noise ratio (S/N) of 3, were 0.1 *μ*g/kg for all analytes. The results show that this method has high sensitivity, good reproducibility, and wide linear range when used in wheat samples determination.

### 3.3. Analysis of Real Samples

**Figure 8:**
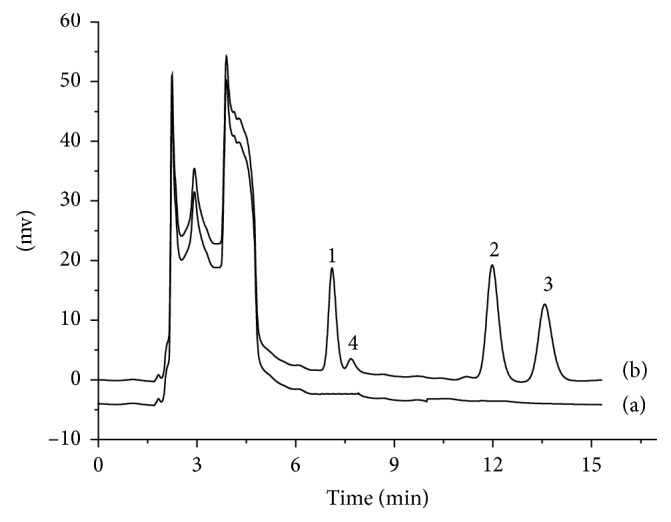
Wheat samples with 5 *μ*g/g concentration of three OPPs were analyzed before (a) and after (b) [OMIM][Tf_2_N]-DLLME. Peaks 1, 2, 3, and 4 represent fenitrothion, fenthion, phoxim, and impurity, respectively.

The method was applied to the wheat samples in three different years. The recoveries of the methods were determined by adding 3 different concentrations of OPPs (1, 5, and 50 *μ*g/g). The results are shown in Table 2. The experimental results show that the recoveries of the three wheat species are between 74.8 and 112.5%, 71.8 and 104.5%, and 83.8 and 115.5%, respectively, which indicates that this method can be used accurately and reliably for the extraction and determination of the actual wheat samples. The chromatograms of the wheat samples adding 5 *μ*g/g of three OPPs before and after extractions are shown in Figure 8. After extracting wheat with methanol, and directly injecting the methanol, the analytes cannot be detected, while after the [OMIM][Tf_2_N]-DLLME process, OPPs can be effectively detected.

### 3.4. Comparison with Other Analytical Methods

To characterize the extraction performance of the proposed method in this work, a comparison of the [OMIM][Tf_2_N] IL-DLLME with other methods is summarized in Table 3. From it, we can see that the advantages of the present method can be described as follows: (i) the amount of the IL used is the least; (ii) the operation time is shorter than most methods; and (iii) the matrix is wheat, and the method shows good linear range and RSD. The results indicate that this method is simple, time-saving, and with satisfactory extraction effect for wheat samples.

## 4. Conclusions

This work firstly proposed a method that used Tf_2_N anion-based ionic liquids (ILs) and dispersive liquid-liquid mircoextraction (DLLME) for extracting OPPs in wheat samples by ultrasonic-assisted and combined with HPLC analysis. By investigating the influencing factors in the extraction process, the optimum conditions were determined. [OMIM][Tf_2_N] ionic liquid exhibited best extraction performance for OPPs among three Tf_2_N-based ILs in the extraction system due to its unique properties. Compared with other methods, the results of this method show that it is a time-saving, simple but efficient method with high sensitivity and reliability. Furthermore, it has good recoveries, wider LRs, and lower LODs, and RSDs indicate that the method is satisfactory for OPPs extraction by using Tf_2_N-based IL as an extractant in wheat samples. It is a promising method to be used for the trace determination of various organic compounds in complex matrices in the future.

## Figures and Tables

**Table 1:** Evaluation of method performance.

Compounds	Linearity range (*μ*g/g)	1 *μ*g/g spiked		5 *μ*g/g spiked		50 *μ*g/g spiked		LOD (*μ*g/kg)
		EF	RSD (%) (*n*=5)	EF	RSD (%) (*n*=5)	EF	RSD (%) (*n*=5)	
Fenitrothion	0.01–100	332.4	1.8	250.4	2.5	203.8	4.6	0.1
Fenthion	0.01–100	251.4	3.8	297.8	5.5	214.3	6.3	0.1
Phoxim	0.01–100	328.4	3.4	265.0	5.9	264.0	0.6	0.1

**Table 2:** Analysis of wheat samples in three different years.

Compounds	Spike level (*μ*g/g)	Wheat in 2002		Wheat in 2006		Wheat in 2010	
		RR (%)	RSD (%) (*n*=3)	RR (%)	RSD (%) (*n*=3)	RR (%)	RSD (%) (*n*=3)
Fenitrothion	1	94.6	4.8	106.8	0.8	99.5	4.2
	5	106.9	3.4	112.5	3.4	100.0	3.7
	50	74.8	1.5	72.9	0.7	83.3	2.8
Fenthion	1	71.8	1.1	90.2	4.2	98.3	3.4
	5	76.9	3.7	74.7	1.8	104.5	3.1
	50	82.7	4.2	74.9	2.8	87.5	3.0
Phoxim	1	115.5	2.3	106.7	3.9	106.9	1.0
	5	83.8	3.2	101.6	5.2	102.3	0.8
	50	108.3	1.8	85.4	3.4	84.1	3.5

**Table 3:** Comparison of this method with previous works.

Method	IL type	IL volume	Process time	Linear range (*μ*g/L) (*μ*g/Kg)	LOD (*μ*g/L) (*μ*g/Kg)	RSD (%)	Matrix	Reference
SBME	[OMIM][PF6]	Not mentioned	>60 min	1–200	<0.026	<3	Water	[29]
LPME	[HMIM][PF6]	50 *μ*L	>50 min	1–100	<0.29	<3	Water	[30]
DLLME	[ODMIM]/[HMIM][Nf2T] mix IL	54 *μ*L	∼13 min	2.5–500	<0.69	<0.69	Fruit juices	[18]
DLLME	[OMIM][PF6]	50 *μ*L	∼6 min	2-100 *μ*g/Kg	<0.73	<5.7	Apple and pear	[28]
DLLME	[OMIM][Tf2N]	22 *μ*L	∼13 min	0.01–100	0.1	<5.9	Wheat	This work

## References

[1] Uygun U., Senoz B., Koksel H. (2008). Dissipation of organophosphorus pesticides in wheat during pasta processing.

[2] Khan I. A. T., Riazuddin, Parveen Z., Ahmed M. (2007). Multi-residue determination of synthetic pyrethroids and organophosphorus pesticides in whole wheat flour using gas chromatography.

[3] Yousefi S. M., Shemirani F., Ghorbanian S. A. (2017). Deep eutectic solvent magnetic bucky gels in developing dispersive solid phase extraction: application for ultra trace analysis of organochlorine pesticides by GC-micro ECD using a large-volume injection technique.

[4] Chormey D. S., Buyukpinar C., Turak F., Komesli O. T., Bakırdere S. (2017). Simultaneous determination of selected hormones, endocrine disruptor compounds, and pesticides in water medium at trace levels by GC-MS after dispersive liquid-liquid microextraction.

[5] Harshit D., Charmy K., Nrupesh P. (2017). Organophosphorus pesticides determination by novel HPLC and spectrophotometric method.

[6] Timofeeva I., Shishov A., Kanashina D., Dzema D., Bulatov A. (2017). On-line in-syringe sugaring-out liquid-liquid extraction coupled with HPLC-MS/MS for the determination of pesticides in fruit and berry juices.

[7] Riazuddin, Khan M. F., Iqbal S., Abbas M. (2011). Determination of multi-residue insecticides of organochlorine, organophosphorus, and pyrethroids in wheat.

[8] Gonzalez-Curbelo M. A., Hernandez-Borges J., Borges-Miquel T. M., Rodríguez-Delgado M. Á. (2013). Determination of organophosphorus pesticides and metabolites in cereal-based baby foods and wheat flour by means of ultrasound-assisted extraction and hollow-fiber liquid-phase microextraction prior to gas chromatography with nitrogen phosphorus detection.

[9] Kevin S. H. W. P., Norman N. T. (2001). Supercritical fluid extraction and quantitative determination of organophosphorus pesticide residues in wheat and maize using gas chromatography with flame photometric and mass spectrometric detection.

[10] Rezaee M., Assadi Y., Hosseinia M. R. M., Aghaee E., Ahmadi F., Berijani S. (2006). Determination of organic compounds in water using dispersive liquid-liquid microextraction.

[11] Sousa R., Homem V., Moreira J. L., Madeira L. M., Alves A. (2013). Optimisation and application of dispersive liquid–liquid microextraction for simultaneous determination of carbamates and organophosphorus pesticides in waters.

[12] Cinelli G., Avino P., Notardonato I., Russo M. V. (2014). Ultrasound-vortex-assisted dispersive liquid–liquid microextraction coupled with gas chromatography with a nitrogen–phosphorus detector for simultaneous and rapid determination of organophosphorus pesticides and triazines in wine.

[13] Farajzadeh M. A., Afshar Mogaddam M. R., Rezaee Aghdam S., Nouri N., Bamorrowat M. (2016). Application of elevated temperature-dispersive liquid-liquid microextraction for determination of organophosphorus pesticides residues in aqueous samples followed by gas chromatography-flame ionization detection.

[14] Pandey S. (2006). Analytical applications of room-temperature ionic liquids: a review of recent efforts.

[15] Marciniak A. (2010). Influence of cation and anion structure of the ionic liquid on extraction processes based on activity coefficients at infinite dilution. A review.

[16] Kudlak B., Owczarek K., Namiesnik J. (2015). Selected issues related to the toxicity of ionic liquids and deep eutectic solvents–a review.

[17] Zhang C., Cagliero C., Pierson S. A., Anderson J. L. (2017). Rapid and sensitive analysis of polychlorinated biphenyls and acrylamide in food samples using ionic liquid-based in situ dispersive liquid-liquid microextraction coupled to headspace gas chromatography.

[18] Zeng H., Yang X., Yang M. (2017). Ultrasound-assisted, hybrid ionic liquid, dispersive liquid-liquid microextraction for the determination of insecticides in fruit juices based on partition coefficients.

[19] Tuzen M., Uluozlu O. D., Mendil D. (2018). A simple, rapid and green ultrasound assisted and ionic liquid dispersive microextraction procedure for the determination of tin in foods employing ETAAS.

[20] De Boeck M., Missotten S., Dehaen W., Tytgat J., Cuypers E. (2017). Development and validation of a fast ionic liquid-based dispersive liquid-liquid microextraction procedure combined with LC-MS/MS analysis for the quantification of benzodiazepines and benzodiazepine-like hypnotics in whole blood.

[21] Faraji M., Noorani M., Sahneh B. N. (2017). Quick, easy, cheap, effective, rugged, and safe method followed by ionic liquid-dispersive liquid-liquid microextraction for the determination of trace amount of bisphenol A in canned foods.

[22] Liu R. Q., Liu Y., Cheng C. S., Yang Y. (2017). Magnetic solid-phase extraction and ionic liquid dispersive liquid-liquid microextraction coupled with high-performance liquid chromatography for the determination of hexachlorophene in cosmetics.

[23] Xia Z., Li D., Li Q., Zhang Y., Kang W. (2017). Simultaneous determination of brazilin and protosappanin B in *Caesalpinia sappan* by ionic-liquid dispersive liquid-phase microextraction method combined with HPLC.

[24] Kowsari M. H., Fakhraee M. (2015). Influence of butyl side chain elimination, tail amine functional addition, and C2 methylation on the dynamics and transport properties of imidazolium-based [Tf_2_N^–^] ionic liquids from molecular dynamics simulations.

[25] Deferm C., Luyten J., Oosterhof H., Fransaer J., Binnemans K. (2018). Purification of crude in(OH)3 using the functionalized ionic liquid betainium bis(trifluoromethylsulfonyl)imide.

[26] Requejo P. F., Calvar N., Domínguez Á., Gómez E. (2016). Application of the ionic liquid tributylmethylammonium bis(trifluoromethylsulfonyl)imide as solvent for the extraction of benzene from octane and decane at T = 298.15 k and atmospheric pressure.

[27] Tagiuri A., Sumon K. Z., Henni A. (2014). Solubility of carbon dioxide in three [Tf_2_N] ionic liquids.

[28] Zhang L., Chen F., Liu S. (2012). Ionic liquid-based vortex-assisted dispersive liquid-liquid microextraction of organophosphorus pesticides in apple and pear.

[29] Zhang Y., Wang R., Su P., Yang Y. (2013). Ionic liquid-based solvent bar microextraction for determination of organophosphorus pesticides in water samples.

[30] Zhou Q., Bai H., Xie G., Xiao J. (2008). Trace determination of organophosphorus pesticides in environmental samples by temperature-controlled ionic liquid dispersive liquid-phase microextraction.

